# Systematic Study of Paeonol/Madecassoside Co-Delivery Nanoemulsion Transdermal Delivery System for Enhancing Barrier Repair and Anti-Inflammatory Efficacy

**DOI:** 10.3390/molecules28135275

**Published:** 2023-07-07

**Authors:** Wangwang Lu, Dan Luo, Dan Chen, Shuting Zhang, Xuan Chen, Hong Zhou, Qian Liu, Siyuan Chen, Wei Liu

**Affiliations:** 1 Guangzhou Jiyan Cosmetics Technology Co., Ltd., Guangzhou 510275, China; leo.lu@yatsenglobal.com (W.L.); jessica.liu@yatsenglobal.com (Q.L.); 2 National Engineering Research Center for Nanomedicine, Huazhong University of Science and Technology, Wuhan 430075, China; laurel565118@163.com (D.L.); yaoxuechendan@163.com (D.C.); 3 College of Life Science and Technology, Huazhong University of Science and Technology, Wuhan 430074, China; m202172340@hust.edu.cn (S.Z.); cx17837172264@163.com (X.C.); hz199268@hust.edu.cn (H.Z.); 4 Institute for Biomaterials, Tech Institute for Advanced Materials, College of Materials Science and Engineering, Suqian Advanced Materials Industry Technology Innovation Center, NJTech-BARTY Joint Research Center for Innovative Medical Technology, Nanjing Tech University, Nanjing 211816, China

**Keywords:** nanoemulsion, transdermal co-delivery, sensitive skin, skin barrier repair, anti-inflammatory

## Abstract

Sensitive skin is defined as skin with low tolerance and high reactivity. Natural products, such as paeoniflorin and madecassoside, have unique skin care functionality. However, because they are hampered by the skin barrier, paeoniflorin and madecassoside have difficulty penetrating the stratum corneum, resulting in weakened skin barrier repair and anti-inflammatory effects. In addition, there is a lack of detailed studies on the efficacy of paeonol and madecassic in human skin, especially in 3D skin models and clinical trials. To overcome the low transdermal delivery issue, we developed nanoemulsions (PM-NEs) loaded with paeonol and madecassoside to improve their delivery efficiency and promote sensitive skin repair and anti-inflammation effects. Furthermore, systematic evaluations of the efficacy in cell line models, 3D skin models, and clinical trials were conducted. The PM-NEs effectively improved the efficacy of paeonol and madecassoside glucoside transdermal penetration and retention and enhanced cellular uptake. Cellular assays and 3D epidermal models showed that the PM-NEs significantly promoted the secretion of filamentous protein, aquaporin 3, Claudin-1, and hyaluronic acid, and considerably inhibited the secretion of interleukin 1α, interleukin 6, tumor necrosis factor-α, and prostaglandin E_2_ compared to free components. Notably, clinical trial data showed that the PM-NEs significantly reduced transepidermal water loss, a* values, erythropoietin, the amount of non-inflammatory acne, and the amount of inflammatory acne in the facial skin. Three levels of systematic studies suggest that co-delivery of paeoniflorin and madecassoside via nanoemulsions is a promising strategy to improve topical delivery efficiency and anti-inflammatory repair efficacy in sensitive skin.

## 1. Introduction

Sensitive skin, which is characterized by low tolerance and high reactivity, is often considered a natural state of the skin’s own structure and sensory nerves, rather than a skin condition [1]. The skin is prone to subjective symptoms, such as burning, tingling, itching, and tightness, when stimulated by physical, chemical, and mental factors. The development of sensitive skin is a gradual, slow-developing process that can progress from mild dryness and tightness to facial flushing, and even to dermatitis, eczema, and other skin diseases [2,3,4,5]. The pathogenesis of sensitive skin is complex and includes impaired skin barrier function, the presence of inflammatory reactions in the epidermis, and abnormalities of the sensory nervous system in the skin. These factors are often mutually reinforcing processes, resulting in a vicious cycle of sensitive skin problems that cannot be easily transformed into normal skin [6,7,8]. Therefore, it is crucial to develop skincare products to improve sensitive skin.

Natural products have unique structural and physiological activity diversity, making them an ideal ingredient source for skincare products [9]. Paeonol, a polyphenolic compound, is an active substance extracted mainly from Moutan cortex and Paeonia lactiflora [10,11]. Several studies have shown that paeonol has numerous biological activities, including antioxidant, anti-inflammatory, antibacterial, analgesic, neuroprotective, etc. [12]. However, its poor water solubility and stability largely impair in vivo bioavailability, thus greatly limiting its application [13]. Madecassoside, a triterpenoid saponin compound isolated mainly from the gotu kola herb (*Centella asiatica*), exhibits a wide range of biological activities, such as antioxidant, anti-apoptotic, anti-inflammatory, neuroprotective, moisturizing, and wound healing effects [14]. It has been applied in the treatment of skin wounds, eczema, and other diseases [15,16]. However, due to its strong polarity, madecassoside has difficulty crossing the skin barrier and penetrating the skin, thus greatly hampering its use in skin care products.

In recent years, nano-carrier technology has been widely applied for the transdermal delivery of drugs and functional cosmetics [17,18]. Various nano-carriers, such as liquid crystalline lipid nanoparticles (LCNPs), nanoemulsions (NEs), etc., have received great research interest. LCNPs could incorporate a variety of hydrophobic and hydrophilic small-molecule drugs and remarkably prolong the drug release profile [19]. For example, Miora et al. developed curcumin, fish oil, and brain-derived neurotrophic factor (BDNF) co-loaded LCNPs for targeting the endoplasmic reticulum to combat neuronal degeneration. The multidrug-loaded LCNPs could significantly improve the BDNF concentration in the target cells compared to BDNF treatment alone, as well as modulate ER stress, demonstrating the therapeutic potential for neuroprotection and the prevention of neurodegeneration [20]. Another nanocarrier technology, nanoemulsions (NEs), offer various advantages over conventional emulsions, such as a large specific surface area, high stability, and adjustable rheology due to their small size (20–200 nm) [21]. NEs are attractive drug delivery systems because of their excellent encapsulation of both hydrophilic and hydrophobic drugs. In addition, by loading drugs into nano-sized particles, the solubility and permeability of drugs are greatly increased, which further leads to enhanced bioavailability and sustained release [22,23,24]. Furthermore, the preparation process of NEs is straightforward and does not require special equipment, which is ideal for industrial mass production. As a result, NEs technology has been widely utilized in cosmetic fields [25,26]. In addition, the co-delivery of drugs with different properties requires the successful development of a carrier capable of containing both drugs. Wang et al. prepared nanoparticles by a double emulsion method incorporating hydrophilic doxorubicin and hydrophobic paclitaxel. The nanocarrier system of two anticancer drugs as a single formulation showed enhanced cellular uptake and simultaneous release of both drugs, resulting in a synergistic anticancer effect of inhibiting tumor cell growth [27]. Rudra et al. developed a w/o/w multiple NE for the co-delivery of pemetrexed (PMX) and quercetin (QCN), and the results demonstrate that the combination of PMX with QCN improved their oral absorption, while providing synergistic anticancer activity. In addition, compared with free PMX and QCN, the oral bioavailability of PMX and QCN multiplex nanoemulsion in rats was 4.51-fold and 23.9-fold, respectively [28]. Therefore, co-delivery of multiple anti-inflammatory and reparative medications via NEs would be a promising strategy for the treatment of sensitive skin.

Given the barrier function of the skin itself, it is difficult for drugs to pass through the stratum corneum to remain in the epidermis and accumulate in the inner part of the skin barrier in an effective concentration range, resulting in a diminished or even absent effect of the drug for the treatment of the skin condition in question. Therefore, it is important to ensure the drug passes through the stratum corneum and enters the active epidermis or dermis to exert its effect. Topical drug delivery methods are of great importance in the treatment of dermatological diseases. In topical drug delivery, penetration enhancement strategies have a crucial impact on the topical delivery of drug nanocarriers [29]. For example, El-Leithy et al. developed a new coenzyme Q10 nanoemulsion with considerably improved skin permeation and a highly prominent anti-wrinkle efficacy [30]. Sharma et al. reported a resveratrol nanoemulsion gel, with experimental results showing that the nanoemulsion gels had stronger penetration and retention effects to prevent UV-induced oxidative skin damage [31]. Herein, we reported a transdermal NE system co-delivery of active anti-inflammatory ingredients, including paeonol and madecassoside (PM-NEs), for the treatment of sensitive skin. The proliferation and migration ability of PM-NEs against immortalized human keratinocytes (HaCaT) were investigated, and the mechanism of barrier repair functionality of the PM-NEs was explored. The anti-inflammatory ability of the PM-NEs was further examined on LPS-induced mouse mononuclear macrophages (RAW264.7). In addition, the soothing effect of the PM-NEs was assessed on SLS-induced three-dimensional epidermal skin models. Finally, a clinical trial was conducted to study the effects of PM-NEs on transepidermal water loss (TEWL), a* value, and red pigment in the skin of sensitive volunteers.

## 2. Results and Discussion

### 2.1. Characterization

**Figure 1:**
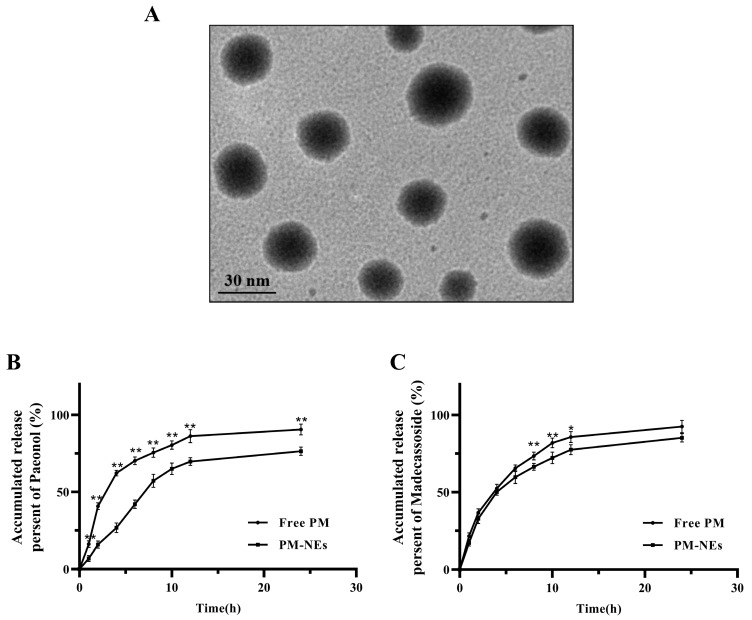
(**A**) TEM image of PM-NEs. The inset shows one representative particle at a higher magnification of one representative particle. In vitro release of paeonol (**B**) and madecassoside (**C**) from the PM-NEs. All data are reported as the mean ± SD (*n* = 3). * *p* < 0.05 ** *p* < 0.01, compared with the PM-Nes group.

The prepared PM-NEs suspension displayed a yellow transparent color. The mean particle size, polydispersity index (PDI), and zeta potential of the PM-NEs were 35.8 ± 0.7 nm, 0.151 ± 0.002, and −28.98 ± 0.20 mV, respectively. As shown in the transmission electron microscopy (TEM) image (Figure 1A), the PM-NEs exhibited a relatively uniform spherical shape with no obvious aggregation observed. The particle size was around 25–35 nm, which was consistent with the particle size measured by the dynamic light scattering method (DLS). The encapsulation efficiency (EE) values of paeonol and madecassoside in the PM-NEs were 88.3 ± 0.4% and 82.3 ± 1.2%, respectively. The drug loading efficiency (LE) values of paeonol and madecassoside were 1.04 ± 0.25% and 1.92 ± 0.18%, respectively.

The stability of the PM-NEs was examined in room temperature, low temperature (4 °C), and high temperature (45 °C) conditions for 30 days. The results show that the particle size and PDI of the PM-Nes were stable under each condition. The PM-Nes remained transparent, and no delamination or emulsion breakage was observed, indicating the good storage stability of PM-NEs.

### 2.2. In Vitro Release

The in vitro release profiles of paeonol and madecassoside in the PM-NEs are shown in Figure 1B,C. Compared with free paeonol, which released 90.6% content after 24 h, only 76.4% of paeonol was released from PM-NEs, suggesting the PM-NEs exhibited noticeable sustained-release effect (Figure 1B). For madecassoside, no obvious improvement in the release profile was observed in the initial 12 h, possibly due to the highly hydrophilic nature of madecassoside [32]. After 24 h, the cumulative release of madecassoside from free madecassoside was as high as 92.5%, and 85.3% madecassoside was released from the PM-NEs (Figure 1C). These results suggest that NEs can significantly prolong the release of paeonol, which is consistent with the reported results on the release of lipophilic drugs from NEs [33]. The prolonged release properties of NEs may be related to the deposition effect of NEs, which can effectively promote the slow release of the loaded active ingredient, thus allowing the loaded drug to exert sustained efficacy over a longer period of time [34].

### 2.3. Transdermal Performance of PM-NEs

**Figure 2:**
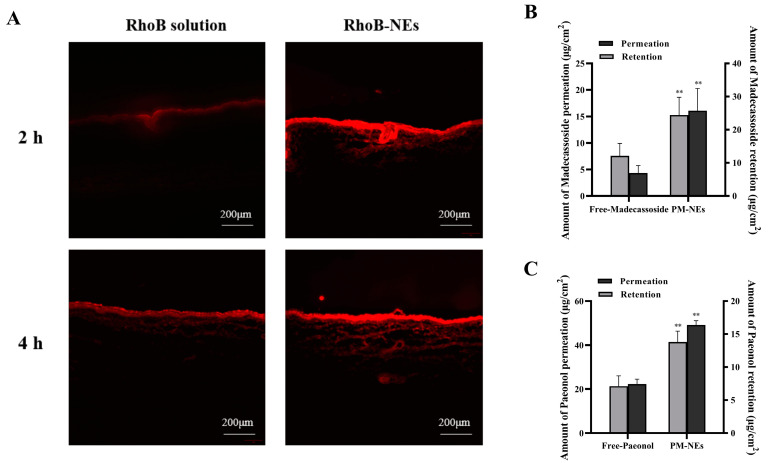
Visualization of RhoB (red fluorescence) delivery pathway in pigskin using a fluorescence microscope during 4 h of permeation in vivo (**A**). Bar graphs representing 24 h cumulative percutaneous permeation and retention of paeonol (**B**) and madecassoside (**C**). All data are reported as the mean ± SD (*n* = 3). ** *p* < 0.01, compared with the free group.

Rhodamine B isothiocyanate (RhoB) was applied as a tracer molecule to investigate the transdermal performance of PM-NEs. As seen in Figure 2A, after 2 h permeation in vivo, the majority of free RhoB was concentrated in the stratum corneum and failed to cross the stratum corneum barrier. In comparison, stronger red fluorescence was observed in pigskin treated with RhoB-NEs, and the deep penetration indicates the RhoB-NEs had already crossed the stratum corneum barrier after a 2 h treatment. By extending the treatment time to 4 h, the fluorescence intensity of the RhoB-NEs was further enhanced and they penetrated the deeper layers of the skin.

The 24 h cumulative percutaneous permeation and retention of the active ingredients were further quantified. Compared with free paeonol, the cumulative skin permeation per unit area of paeonol in the PM-NEs increased by 121.2%, and the skin retention increased by 94.4% (Figure 2B). Compared with free madecassoside, the cumulative skin permeation per unit area of madecassoside in the PM-NEs increased by 273.5%, and skin retention increased by 102.4% (Figure 2C). These results demonstrate that the developed PM-NEs greatly improved the percutaneous permeability and storage capacity of paeonol and madecassoside in the skin. The stratum corneum of healthy skin is an effective barrier against the absorption of active compounds. Nanoemulsions with small particle size can exhibit a good closure effect on the skin and enhance skin affinity, thus facilitating the penetration of the active ingredients through the stratum corneum into the deeper skin layers [35]. Moreover, according to a previous report, NEs with a smaller size showed improved penetration into the skin. The particle size of our PM-NEs is less than 100 nm, which is a suitable size for skin surface adhesion and stratum corneum penetration [36].

### 2.4. Cell Safety Evaluation

**Figure 3:**
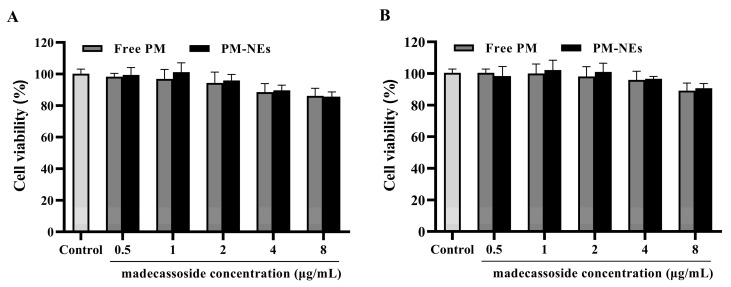
In vitro cytotoxicity of PM-NEs against HaCaT cells (**A**) and RAW264.7 cells (**B**). The cells were treated with the free-PM or PM-NEs at madecassoside concentrations over a wide range (0.5~8 μg/mL). The cells treated with DMEM only served as the control group. All data are reported as the mean ± SD (*n* = 3).

As shown in Figure 3A,B, after treating immortalized human keratinocytes (HaCaT) and mouse mononuclear macrophage (RAW264.7) cells with free PM or PM-NEs within the madecassoside concentration range of 0.5~8 μg/mL for 24 h, the cell viabilities all remained above 80%, suggesting that almost no cytotoxicity was observed. These results confirm that the PM-NEs were safe for both cells in the above-mentioned concentration range. A relatively safe concentration range (0.5 to 8 μg/mL) was selected based on cell safety evaluation, considering that cell activity decreases at an active concentration of 8 μg/mL, and at a concentration of 0.5 μg/mL, the concentration is too low and may not reach the onset of action concentration. Therefore, the middle three concentrations within the safety concentration range were chosen for further experiments.

### 2.5. Cellular Uptake Research

**Figure 4:**
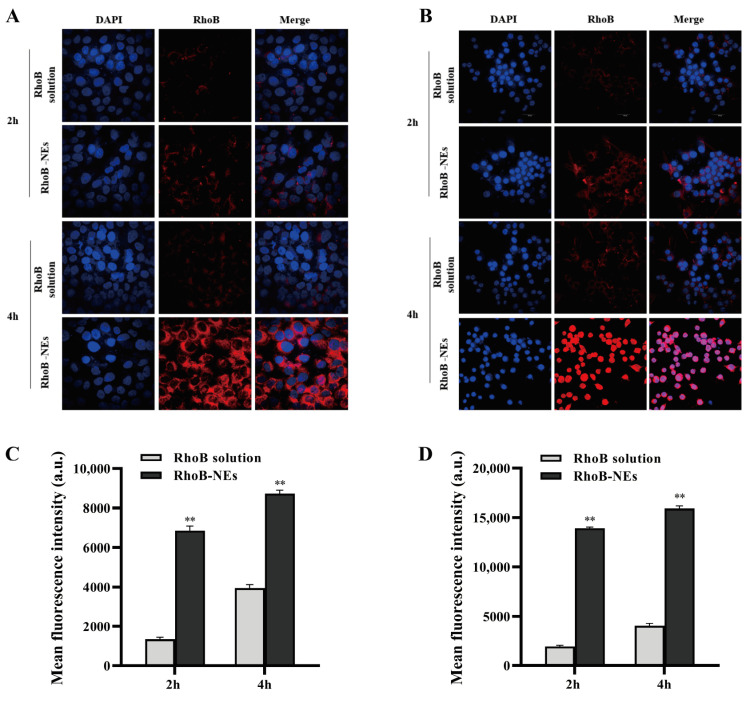
CLSM images of HaCaT cells (**A**) and RAW264.7 cells (**B**) showing the cellular uptake of RhoB-NEs. The cells were treated with 2 μg/mL free RhoB and an equal RhoB concentration of RhoB-NEs for 2 h or 4 h. DAPI was utilized to label the nucleus. Cellular uptakes of RhoB by HaCaT cells (**C**) and RAW264.7 cells (**D**) were further analyzed by FCM and presented as relative mean fluorescence intensity. The FCM samples were analyzed after treating cells with free RhoB or RhoB-NEs at a fixed RhoB dosage of 2 μg/mL for 2 h or 4 h. All data are reported as the mean ± SD (*n* = 3). ** *p* < 0.01, compared with the RhoB solution group.

To assess the uptake behavior of NEs by target cells, the cellular uptake of the NEs was investigated by two cellular models, HaCaT and RAW264.7, which are commonly used as cellular models for assessing skin barrier repair and anti-inflammatory effects [37,38]. The fluorescent dye RhoB was encapsulated in the NEs and the cellular uptake of the NEs was analyzed by flow cytometry (FCM) and confocal laser scanning microscope (CLSM). As shown in the CLSM images, the fluorescence intensity of RhoB in both the HaCaT cells (Figure 4A) and RAW264.7 cells (Figure 4B) treated with RhoB-loaded Nes was substantially higher than that of cells treated with the RhoB solution at the same concentration. The enhanced cellular uptake by the NEs was further quantified by FCM analysis. The mean fluorescence intensities of the HaCaT cells (Figure 4C) and RAW264.7 cells (Figure 4D) treated with RhoB-NEs for 2 h were 4.04 and 6.12 times higher, respectively, than those treated with the RhoB solution. The results of CLSM and FCM demonstrate that NEs can effectively deliver drugs into various relevant cells and significantly improve the intracellular accumulation of drugs.

### 2.6. Cell Proliferation and Migration Assay

**Figure 5:**
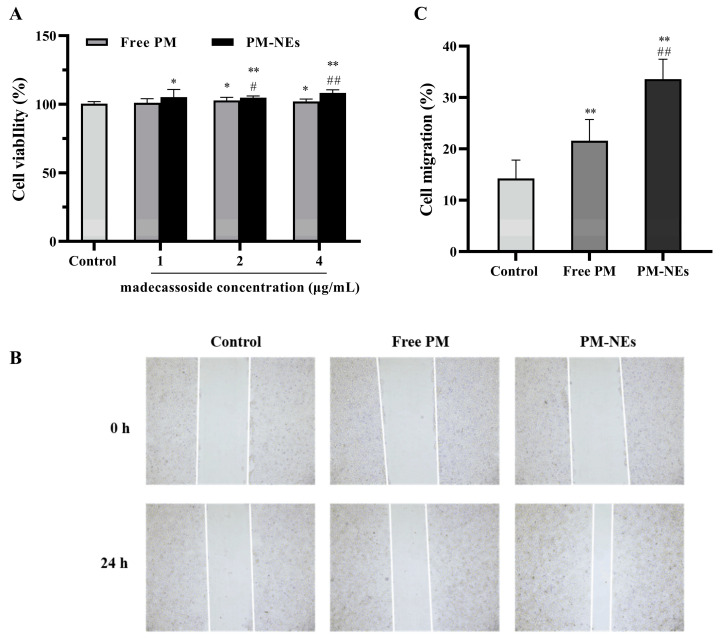
The effect of PM-NEs on the proliferation of HaCaT cells (**A**). The cells were treated with the free PM or PM-NEs at a madecassoside concentration of 1, 2, or 4 μg/mL for 48 h. Evaluation of the effect of PM-NEs on the migration of HaCaT with a scratch migration assay (**B**) and the cell migration rate in the corresponding experiment (**C**). HaCaT cells were treated with free PM or PM-NEs at madecassoside concentration of 2 μg/mL for 24 h. The cells treated with DMEM only served as the control group. All data are reported as the mean ± SD (*n* = 3). * *p* < 0.05, ** *p* < 0.01, compared with the control group; # *p* < 0.05, ## *p* < 0.01, compared with the free PM group.

The HaCaT were treated with free PM and PM-NEs at various concentrations for 48 h. As illustrated in Figure 5A, compared with free PM, the PM-NEs significantly enhanced the cellular viability of HaCaT cells within the madecassoside concentration range of 1–4 μg/mL, implying that the PM-NEs could better promote cell proliferation compared with free PM.

Cell migration is an important property of HaCaT cells in skin repair [39]. To evaluate the effect of PM-NEs on cell migration, the cell migration ability was assessed in HaCaT using a scratch assay. As shown in Figure 5B,C, after incubation for 24 h, the wound distance was significantly reduced in both the free PM and PM-NEs groups compared to the distance in the control group (*p* < 0.05). Furthermore, the wound distance in the PM-NEs group was significantly shorter than that in the free PM group (*p* < 0.01), indicating that PM-NEs could facilitate the migration of HaCaT cells.

### 2.7. Skin Barrier Repair Study

**Figure 6:**
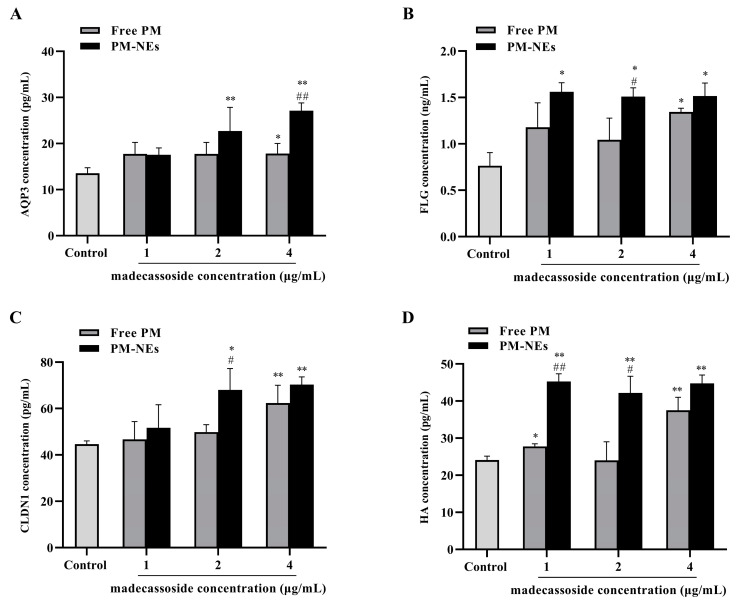
Effects of PM-NEs on the secretion of AQP3 (**A**), FLG (**B**), CLDN1 (**C**), and HA (**D**) in HaCaT cells. Cells were treated with free PM or PM-NEs at a madecassoside concentration of 1, 2, or 4 μg/mL for 48 h. All data are reported as the mean ± SD (*n* = 3), * *p* < 0.05, ** *p* < 0.01, compared with the control group. # *p* < 0.05, ## *p* < 0.01, compared with the free PM group.

Aquaporin (AQP) is a transporter protein that transports small molecules across the cell membrane and is the most expressed water channel protein isoform in human skin [40,41]. AQP3 transports water, glycerol, and triglycerides to the epidermis and maintains epidermal moisture, which has a positive effect on skin barrier repair and moisturization [42]. Therefore, the secretion of AQP3 in HaCaT cells was assessed after treatment with free PM or MPH-NEs. As shown in Figure 6A, the secretions of AQP3 in the HaCaT cells treated with PM-NEs at a certain madecassoside concentration for 48 h were all significantly increased in comparison with the control group. At a madecassoside concentration of 4 μg/mL, the secretion of AQP3 from the PM-NEs was remarkably increased by 52.2% in comparison with the free PM (*p* < 0.01).

Filaggrin (FLG) plays a crucial role in the barrier function and skin moisturization. FLG promotes epidermal differentiation. With the assistance of FLG monomer linkage, keratin fibril bundles aggregate regularly, which, in turn, causes a tight cellular state and forms a barrier structure in the epidermal stratum corneum. The degradation of FLG can form a natural moisturizing factor [43,44]. As shown in Figure 6B, remarkably increased FLG secretion was observed in the HaCaT cells treated with PM-NEs at various concentrations for 48 h compared with the control group. At a madecassoside concentration of 2 μg/mL, the secretion of FLG from the PM-NEs was considerably increased by 44.6% compared with the free PM (*p* < 0.05).

Tight junction proteins (TJP), such as the Claudin family proteins, are essential for maintaining the selective permeability barrier function of epidermal cells. Claudin-1 (CLDN1) is the main transmembrane protein forming the parietal epidermal barrier in TJP [45,46,47]. As shown in Figure 6C, compared with the control group, the secretion of CLDN1 after treatment with PM-NEs at a certain madecassoside concentration for 48 h was considerably enhanced. At a madecassoside concentration of 2 μg/mL, 36.6% increased secretion of CLDN1 was observed in the PM-NEs than the free PM (*p* < 0.05).

Hyaluronic acid (HA) is a major component of the extracellular matrix and synovial fluid, which affects intracellular functions, including the regulation of cell adhesion and cell growth and differentiation; it also promotes HaCaT cell reconstruction and repair. Moreover, HA has water-absorbing and water-holding properties [48,49,50]. Thus, the secretion of HA by the HaCaT cells after PM-NEs treatment was evaluated. As shown in Figure 6D, after treatment with PM-NEs at various madecassoside concentrations for 48 h, the secretion of HA was notably improved in comparison with the control group (*p* < 0.05). At a madecassoside concentration of 1 μg/mL, the secretion of HA from the PM-NEs was significantly enhanced by 75.5% than the free PM (*p* < 0.01).

These data suggest that PM-NEs could repair the skin’s barrier function by increasing the secretion of AQP3, FLG, CLDN1, and HA in HaCaT cells, which was more effective than the free PM.

### 2.8. Cellular Anti-Inflammatory Study

**Figure 7:**
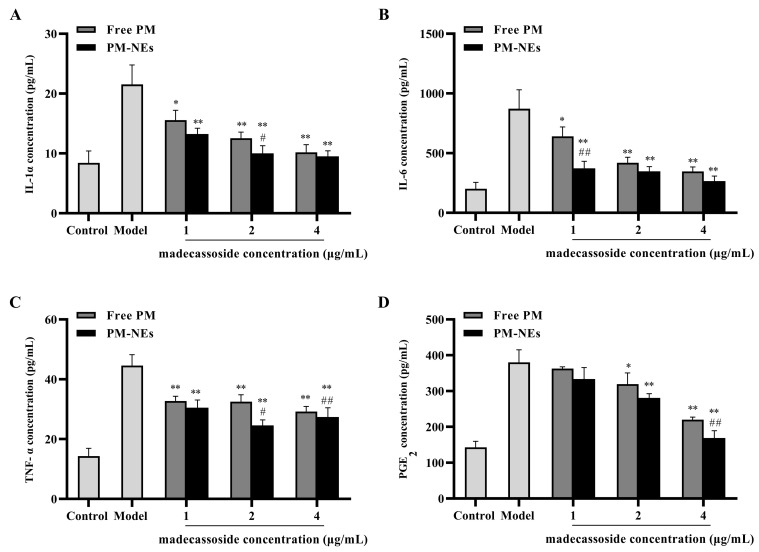
Effects of PM-Nes on the secretion of IL-1α (**A**), IL-6 (**B**), TNF-α (**C**), and PGE_2_ (**D**) in RAW264.7 cells. Cells were treated with free PM or PM-NEs at a madecassoside concentration of 1, 2, or 4 μg/mL for 48 h. All data are reported as the mean ± SD (*n* = 3), * *p* < 0.05, ** *p* < 0.01, compared with the model group. # *p* < 0.05, ## *p* < 0.01, compared with the free PM group.

To further explore the anti-inflammatory mechanism of PM-NEs, the expression levels of pro-inflammatory factors mouse interleukin 1α (IL-1α), interleukin 6 (IL-6), tumor necrosis factor-α (TNF-α), and prostaglandin E_2_ (PGE_2_) in the supernatant of LPS-induced RAW264.7 cells were investigated. Free PM and PM-NEs significantly decreased the protein level of IL-1α (Figure 7A), IL-6 (Figure 7B), TNF-α (Figure 7C), and PGE_2_ (Figure 7D) in LPS-induced RAW264.7 cells, indicating that both the free PM and PM-NEs had anti-inflammatory effects (*p* < 0.05). Compared with free PM, the PM-NEs significantly decreased the protein levels of IL-1α (Figure 7A), IL-6 (Figure 7B), TNF-α (Figure 7C), and PGE_2_ (Figure 7D), indicating that the anti-inflammatory action of the PM-NEs was higher than those of free PM at a certain madecassoside concentration (*p* < 0.05). These results suggest that PM-NEs could attenuate inflammation by reducing the pro-inflammatory factor content at the cellular level.

### 2.9. 3D Skin Model Barrier Repair and Anti-Inflammatory Study

**Figure 8:**
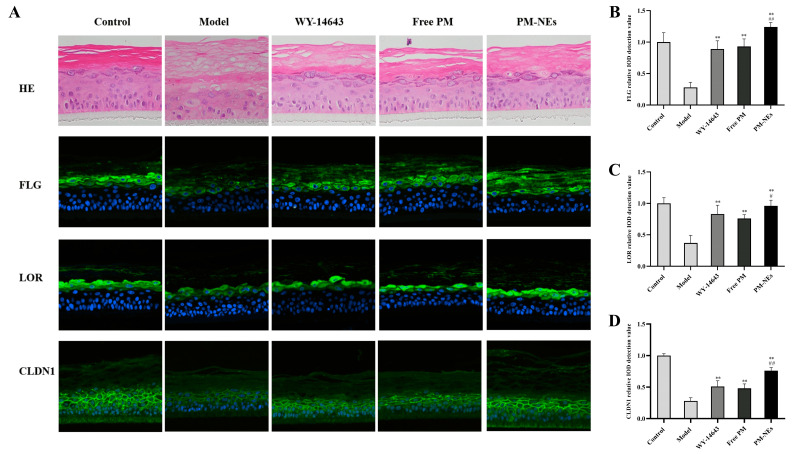
The barrier repair effects of PM-NEs studied using 3D epidermal skin models. The H&E staining and fluorescent immunization images of FLG, LOR, and CLDN1 in the 3D skin model (**A**). The relative value of synthesized FLG (**B**), LOR (**C**), and CLDN1 (**D**) were quantified by immunofluorescence microscopy. All data are reported as the mean ± SD (*n* = 3). ** *p* < 0.01, compared with the model group. # *p* < 0.05, ## *p* < 0.01, compared with the free PM group.

A 3D skin model is an efficient skin model with biochemical signals and mechanical and structural properties that are almost identical to the in vivo physiological state compared to conventional two-dimensional models [51]. Here, we investigated the barrier repair and anti-inflammatory efficacy of PM-NEs using a 3D skin model to simulate human skin. As shown in Figure 8A, compared with the normal group, the epidermal model had a loosened and thickened stratum corneum, a damaged live cell layer, a reduced number of live cell layers, and the appearance of vacuoles (red circles), indicating that the stimulation conditions were effective. Compared with the model group, the free PM group and PM-NEs group showed a thickening of the living cell layer and a significant improvement of the stratum corneum laxity, signifying that the barrier repair effect of the PM-NEs was stronger than that of the free PM. In addition, the immunofluorescence results reveal that stronger green fluorescence from FLG, loricrin (LOR), and CLDN1 were detected in the 3D skin treated with PM-NEs than the model group (Figure 8A), demonstrating that the 3D skin treated with PM-Nes synthesized more FLG, LOR, and CLDN1. The ensembles of FLG (Figure 8B), LOR (Figure 8C), and CLDN1 (Figure 8D) in the PM-NEs group were significantly increased by 342.8%, 159.5%, and 171.4%, respectively, compared with the model group (*p* < 0.01). Notably, the ensemble of FLG, LOR, and CLDN1 in the 3D skin model treated with PM-Nes remarkably increased by 33.3%, 26.3%, and 58.2%, respectively, compared with the free PM (*p* < 0.05). Overall, these results demonstrate that the PM-NEs were also effective in enhancing the barrier repair effect of the active ingredients in the 3D skin models.

**Figure 9:**
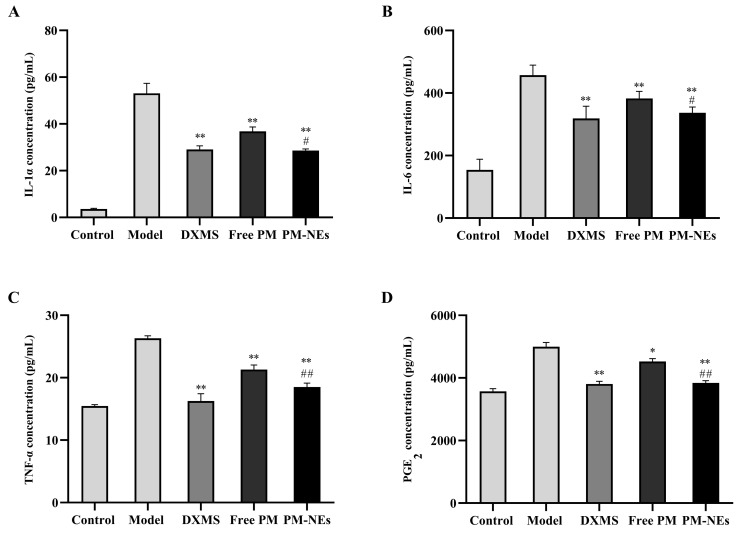
The soothing and anti-inflammatory effects of PM-NEs studied using 3D epidermal skin models. Effects of PM-NEs on the secretion of IL-1α (**A**), IL-6 (**B**), TNF-α (**C**), and PGE_2_ (**D**) in the 3D epidermal skin model. All data are reported as the mean ± SD (*n* = 3). * *p* < 0.05, ** *p* < 0.01, compared with the model group. # *p* < 0.05, ## *p* < 0.01, compared with the free PM group.

Similar to LPS-induced RAW264.7 secreting inflammatory factors, the levels of inflammatory factors (IL-1α, IL-6, TNF-α, and PGE_2_) were significantly increased in the model group after SLS induction, and the expression of inflammatory factors was drastically suppressed after applying PM-NEs to the 3D skin model (Figure 9), indicating that the PM-NEs had a soothing and anti-inflammatory effect. These results suggest that the PM-NEs suppressed the skin inflammatory response by improving the epidermal structure and decreasing the levels of inflammatory factors.

### 2.10. Clinical Study of the Skin Repair and Relief Effect of PM-NEs

**Figure 10:**
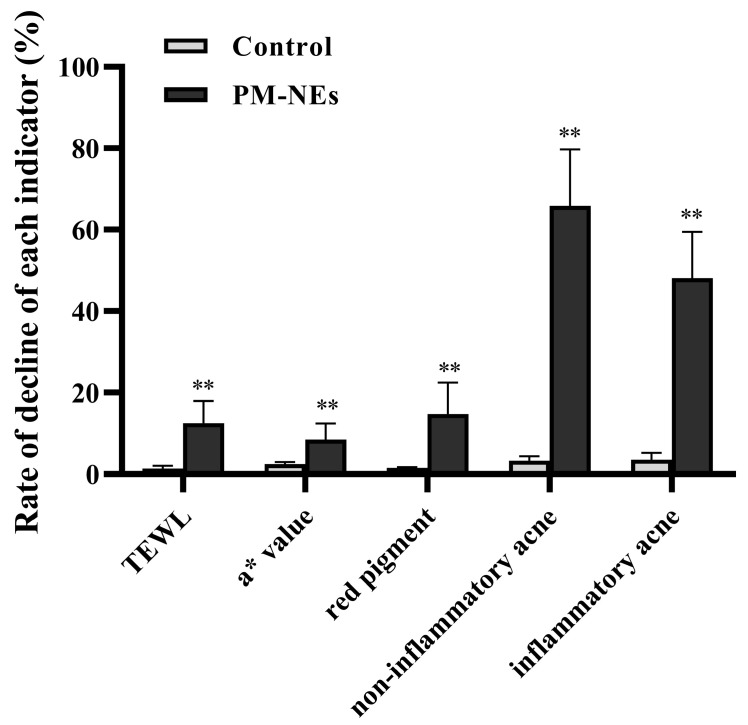
Effect of PM-NEs on TEWL, a* value, red pigment, non-inflammatory acne, and inflammatory acne in a clinical trial. All data are reported as the mean ± SD (*n* = 34). ** *p* < 0.01, compared with the control group.

To evaluate the mildness of the product (an essence containing 5% PM-NEs), a clinical trial was conducted by applying the product on the faces of 34 volunteers. The results of the instrumental measurement and the investigator’s clinical evaluation are shown in Figure 10. No case of Grade 1 or higher adverse skin reactions to the test product was observed during the 28-day trial period, which is evidence of a good safety profile. After using this product for 4 weeks, the transepidermal water loss (TEWL) value of the cheek of the side of the face that was tested in the PM-NEs group decreased by 12.5%, significantly higher than the control group (*p* < 0.01), indicating that the product containing 5% PM-NEs had a restorative effect. After 4 weeks, the a* values and red pigment of a post-acne erythematous area in the PM-NEs group decreased by 8.55% and 14.8%, respectively, notably higher than the control group (*p* < 0.01), indicating that the product exhibited a fading and soothing effect on post-acne erythematous areas. In addition, after 4 weeks, the PM-NEs group showed a 65.89% reduction in non-inflammatory acne (number of blackheads and whiteheads) and a 48.10% reduction in inflammatory acne (number of red papules), considerably higher than the control group (*p* < 0.01), implying that the product exhibited good efficacy in eliminating acne. The most important function of the skin is to form an effective barrier between the “inside” and the “outside” of the body. The stratum corneum, which is the main physical barrier, regulates the evaporation of water from inside the body. TEWL, which is the amount of water that passively evaporates through the skin into the external environment, is used to characterize the skin barrier function [52]. In previous studies, the pharmacodynamic studies on paeoniflorin and madecassoside were mainly focused on the cellular-molecular level, while studies on three-dimensional skin models and clinical trials were limited. Due to skin’s complex structure and high internal coordination, single-cell models cannot fully reflect the drug–skin interaction. Therefore, we not only verified the barrier repair and anti-inflammatory efficacy of PM-NEs against SLS stimulation by 3D skin models based on cellular experiments, but we also conducted human clinical trials to demonstrate the therapeutic effectiveness of PM-NEs to improve the condition of sensitive skin in humans. The clinical trial data shows that the PM-NEs significantly reduced the transepidermal water loss, a* values, red pigmentation, and the number of non-inflammatory acne and inflammatory acne in facial skin. These clinical results suggest that the transdermal combination of nanocarrier technology can enhance the barrier repair effect of paeoniflorin and aristolochia glycosides; they also indicate good mildness of products with high PM-NEs concentration (5%) and good efficacy in repairing sensitive skin, eliminating acne, and fading red acne marks. This also provides a new strategy for the development of skin barrier repair and anti-inflammatory skincare products for sensitive skin.

## 3. Materials and Methods

### 3.1. Materials

The paeonol was purchased from Huike Plant (Xian, Shanxi, China); the madecassoside was purchased from Seppic (Paris, France); the pentanediol was purchased from B&B (Seoul, Republic of Korea); the hexanediol was purchased from Inolex (Philadelphia, PA, USA); the trioctyl/capric glyceride was purchased from Croda (Yorkshire, UK); and the polyglyceryl-6 poly (ricinoleic acid) and polyglycerol-4 oleate was purchased from sungwoo Chemical (Tokyo, Japan).

Fetal bovine essence (FBS), Dulbecco’s modified Eagle’s medium (DMEM), phosphate-buffered saline (PBS, pH 7.4), penicillin, streptomycin, and trypsin-EDTA were purchased from Gibco (Invitrogen, Carlsbad, CA, USA). 4′,6-diamidine-2′-phenylindole dihydrochloride (DAPI) and rhodamine B isothiocyanate (RhoB) were purchased from Sigma Aldrich (St. Louis, MO, USA).

The cell counting kit-8 (CCK-8) kit was obtained from Dojindo (Kumamoto, Japan). The AQP3, FLG, CLDN1, and HA ELISA kits and mouseIL-1α, IL-6, TNF-α, and PGE_2_ ELISA kits were purchased from Jiangsu Mei Mian Industry Co., Ltd. (Nanjing, China).

### 3.2. Cell Culture

HaCaT (Kunming Cell Bank of Chinese Academy of Sciences) and RAW264.7 (Kunming Cell Bank of Chinese Academy of Sciences) were cultured in DMEM with 10% FBS and 1% penicillin/streptomycin. The cells were incubated in a humidified atmosphere with 5% CO_2_ at 37 °C.

### 3.3. Preparation and Characterization of the PM-NEs

The PM-NEs were prepared using a high-pressure homogenization technique. Briefly, 1% (*w*/*w*) paeonol, 3% (*w*/*w*) trioctyl/capric glyceride, 3% (*w*/*w*) polyglyceryl-4 oleate, and 5% (*w*/*w*) polyglyceryl-6 poly(ricinoleic acid) were mixed to produce phase A. Phase B was produced by mixing 13% (*w*/*w*) pentanediol and 8% (*w*/*w*) hexanediol. Then, 2% (*w*/*w*) madecassoside was dissolved in distilled water to give phase C. Subsequently, phases A and B were added to phase C and stirred for a further 45 min at 45 °C, followed by homogenization using an AMH-3 microjet high pressure homogenizer (Antos Nanotechnology, Suzhou, China) at 800 bar 3 times. Finally, the PM-NEs sample was purified by ultrafiltration (MWCO 3.5 kDa, Amicon Ultra, Millipore, Billerica, MA, USA) at 4000× *g* for 30 min. The RhoB-loaded NEs (RhoB-NEs) were prepared following the same method described above, except that RhoB was added to phase C to replace the active ingredient.

The particle size, PDI, and zeta potential of the PM-NEs were measured by dynamic light scattering (DLS) using a Zetasizer/Nano-ZS90 instrument (Malvern Instruments, Malvern, UK). The morphology of the PM-NEs was determined using transmission electron microscopy (TEM, HT7700, Hitachi, Tokyo, Japan). The PM-NEs were diluted 400 times with deionized water, dropped on the copper grid, stained by 1% phosphomolybdic acid, and air-dried before observation by TEM [53].

The LE and EE of the PM-NEs were measured by an ultrafiltration–centrifugation method. The content of paeonol and madecassoside in the samples was analyzed using a BOCL 101 high-performance liquid chromatography (HPLC) system (Shimadzu Instruments, Columbia, MD, USA) with a ChromCore AR C18 column (4.6 mm × 250 mm, 5.0 µm, Suzhou, China) at a wavelength of 262 nm, 30 °C. The mobile phase was an acetonitrile: ultrapure water solution = 10:90 (*v*:*v*), the elution time was around 15 min, the flow rate was 1.0 mL/min, and the injection volume was kept at 10 μL. The LE and EE of the PM-NEs were calculated using the following equations:

$$\mathrm{LE}\left(\%\right)=\frac{\mathrm{We}}{\mathrm{Wm}}\times 100$$

$$\mathrm{EE}\left(\%\right)=\frac{\mathrm{We}}{\mathrm{We}+\mathrm{Wf}}\times 100$$

In which We is the mass of the active ingredients encapsulated in the nanocarrier, Wm is the total mass of the nanocarrier, and Wf is the mass of the free active ingredients not encapsulated in the nanocarrier.

The prepared PM-NEs were stored in brown sealed volumetric flasks at room temperature, low temperature (4 °C), and high temperature (45 °C), respectively. The particle size and PDI were measured after 7, 15, and 30 days.

### 3.4. In Vitro Release Study

The in vitro release behaviors of paeonol and madecassoside from the PM-NEs were investigated by dialysis. Dialysis tubing (MWCO 3500 Da) was boiled in ultrapure water for 5 min and soaked in a 50% ethanol solution overnight before use. Approximately 2 mL of the PM-NEs and an equal volume and concentration of the free PM suspension were loaded into the dialysis tubing and immersed in 80 mL of release medium (PBS solution containing 20 *v*/*v*% propylene glycol, pH 7.4). The release study was conducted in a shaker at 100 rpm, 37 °C for 24 h. One milliliter of the sample was withdrawn after 1, 2, 4, 6, 10, 12, and 24 h and immediately supplemented with the same volume of release medium. The concentrations of paeonol and madecassoside in the samples were determined by HPLC and the cumulative release of paeonol and madecassoside from the PM-NEs was calculated.

### 3.5. In Vitro Skin Permeation

Skin tissue was taken from the backs of BAMA miniature pigs (5–6 kg body weight) with undamaged hair follicles, purchased from Zhifu Yurong Biological Studio (Yantai, Shandong, China). The transdermal properties of the PM-NEs were investigated using the Franz diffusion cell method. The BAMA pigskin was fixed between the supply pool and the receiving pool, with the stratum corneum oriented toward the supply pool. A total of 0.5 mL of the PM-NEs and 0.5 mL of the free PM containing the same concentration of PM were added to the supply pool and applied uniformly to the BAMA pigskin, respectively. The Franz diffusion cells were maintained at 37 °C using a recirculating water bath, and the fluid in the receptor chambers was stirred continuously at 300 rpm. At various time intervals (1, 2, 4, 6, 8, 10, 12, and 24 h), a 0.5 mL sample was withdrawn from the receiving pool and replenished with an equal volume of fresh receiving solution at the same temperature. After 24 h, the skin was removed and ground with 2 mL methanol, ultrasonicated for 10 min, and then centrifuged. The supernatant was filtered and used for HPLC analysis to calculate the retention of the active ingredients per unit area of skin.

To visualize the transdermal permeation process of the PM-NEs, 0.5 mL of RhoB-NEs or free RhoB solution with the same RhoB concentration were added to the supply pools, respectively, and applied uniformly to the BAMA pigskin. During percutaneous permeation, the skin was removed at different time points (2 h and 4 h) and skin tissue sections were prepared using a cryotome (Thermo Scientific, HM525NX, Shanghai, China). The distribution of RhoB in the skin tissue sections was observed using fluorescence microscopy (IX71, Olympus, Tokyo, Japan).

### 3.6. In Vitro Cytotoxicity

The HaCaT and RAW264.7 cells were seeded separately in 96-well plates at a density of 1 × 10^4^ or 1.5 × 10^4^ cells per well, respectively. After being cultured for 24 h at 37 °C, the cells were treated with free PM or PM-NEs at a madecassoside concentration of 0.5, 1, 2, 4, and 8 μg/mL. The cells treated with 100 μL DMEM only were used as a control. After incubation for 24 h, the cells were washed with PBS and treated with 100 μL of 10% (*v*/*v*) CCK-8 solution, and the cell viability of the HaCaT and RAW264.7 cells were measured after 2 h of incubation at 37 °C with 5% CO_2_.

### 3.7. Cellular Uptake Study

The uptake of the NEs by the HaCaT and RAW264.7 cells was visualized using confocal microscopy. The HaCaT and RAW264.7 cells were seeded in 35-mm glass bottom dishes at a density of 2 × 10^5^ cells per dish and incubated for 24 h, then were treated with the free RhoB solution (2 μg/mL) or RhoB-NEs with the same RhoB concentration and incubated for 2 h and 4 h. Subsequently, the cells were washed three times with cold PBS and fixed with 4% paraformaldehyde in PBS for 15 min. After fixation, the cells were treated with DAPI solution (5 μg/mL) to label the nucleus and imaged by a confocal laser scanning microscope (CLSM, Olympus, FV3000, Tokyo, Japan) with an excitation wavelength of 405 nm and 561 nm, respectively.

The uptake of the NEs by the HaCaT and RAW264.7 cells was further quantitatively analyzed by flow cytometry. The HaCaT and RAW264.7 cells were seeded in 6-well plates at a density of 3 × 10^5^ cells per well, separately, and incubated for 24 h, then were treated with the RhoB-NEs or free RhoB solution with the same RhoB concentration (2 μg/mL) for 2 h or 4 h. After co-incubation, the culture medium was removed. The treated cells were washed with cold PBS, trypsinized, centrifuged, and resuspended in 0.6 mL of cold PBS prior to flow cytometry analysis (FC500, Beckman Coulter, Fullerton, CA, USA) with an excitation wavelength of 561 nm.

### 3.8. Cellular Proliferation and Migration Assay

The proliferative effect of the PM-NEs on the HaCaT cells was assessed by CCK-8. The HaCaT cells were seeded in 96-well plates at a density of 1 × 10^4^ cells per well. After culturing for 24 h at 37 °C, the culture medium was replaced by free PM or PM-NEs at a madecassoside concentration of 1, 2, or 4 μg/mL for 48 h. The cells treated with DMEM only were used as a control.

The HaCaT cells were seeded in 6-well plates and grown to 90% confluence in DMEM medium containing 10% FBS. Straight lines were scratched on the cells in each well using 200 μL sterile pipette tips, followed by washing three times with sterile PBS to remove cell debris. Next, the HaCaT cells were treated with free PM or PM-NEs at a madecassoside concentration of 2 μg/mL. Photographs of the wounded area were taken under the microscope (MshOt, MF52-N, Guangzhou, China) at 0 h and 24 h after treatment.

### 3.9. Cellular Barrier Repair Study

The HaCaT cells were seeded in 24-well plates at a density of 8 × 10^4^ cells per well and cultured for 24 h. Next, the cells were treated with free PM or PM-NEs at a madecassoside concentration of 1, 2, or 4 μg/mL. The cells treated with DMEM only were used as a control. After incubation for 24 h, the content of FLG, AQP3, CLDN1, and HA in the medium was determined using the human FLG, AQP3, CLDN1, and HA ELISA Kit, respectively.

### 3.10. Cellular Anti-Inflammatory Study

The RAW264.7 cells were seeded in 24-well plates at a density of 8 × 10^4^ cells per well and cultured for 24 h. Next, the cells pretreated with 1 μg/mL LPS were incubated with free PM or PM-NEs at a madecassoside concentration of 1, 2, or 4 μg/mL for 24 h. The HaCaT cells treated with DMEM were utilized as the control group and the cells treated with 1 μg/mL LPS for 24 h were utilized as the model group. After incubation for 24 h, the supernatant was collected and the levels of TNF-α, IL-1β, IL-6, and PGE_2_ were determined using ELISA kits according to standard protocols.

### 3.11. Three-Dimensional Skin Model Efficacy Study

The commercially available epidermal tissue EpiKutis^®^ (Guangdong Biocell Biotechnology Co., Ltd., Dongwan, China) is an in vitro reconstructed human epidermis (RHE) that is histologically similar to in vivo human epidermis [54]. The EpiKutis^®^ models were removed from nutrient agar and equilibrated overnight in 6-well plates at 37 °C with 5% CO_2_ and ~95% relative humidity. The 3D skin model was randomly divided into the control group, model group, positive control group, free PM group, and PM-NEs group, with three replicates in each group. For the free PM and PM-NEs groups, the EpiKutis^®^ was first stimulated with 12.5 μL of 0.2% SLS and then treated with the same volume of free PM and PM-NEs (1% PM-NEs, *w*/*w*) for 24 h. In the study, EpiKutis^®^ treated with 12.5 μL of pyrantel (WY-14,643, 50 μM) was chosen as a positive control for the barrier repair effect and 12.5 μL of dexamethasone (DXMS, 250 μM) as a positive control for the anti-inflammatory effect. Unstimulated skin was used as a blank control and 0.2% SLS-stimulated skin was used as a negative control. After incubation for 24 h, residue on the surface of the 3D skin tissue was washed with sterile PBS solution, fixed with 4% formaldehyde, embedded in paraffin, and cut into thin slices. The slices were stained with hematoxylin and eosin (H&E) for histological analysis. The models used for the assay were treated with 4% paraformaldehyde for fixation, and after 24 h fixation, immunofluorescence of the FLG, loricrin (LOR), and CLDN1 were detected. Pictures were taken and observed under the microscope, and the pictures were collected and analyzed. The concentration of IL-1α, IL-6, TNF-α, and PGE_2_ in the supernatant of the lower chamber was detected by ELISA.

### 3.12. Clinical Trial

To investigate the clinical efficacy of the PM-NEs in removing acne, repairing skin, and soothing sensitive skin, 2 males and 32 females between the ages of 18 and 50, with mild or moderate post-acne erythema on their faces, sensitive skin, and positive lactic acid sting test (total score ≥ 3) were selected as volunteers. Informed written consent was obtained from the human subjects under a protocol approved by the Institutional Ethics Committee of the Third Affiliated Hospital of Zhongshan Medical University (ethical approval number: Medical Ethics Committee 2020-008-01). Two areas of each subject’s face were randomly selected as either test or control. The test areas were treated with an essence containing 5% PM-NEs (*n* = 34), while the control areas received a blank essence (*n* = 34), and a blank cream was used as a placebo. After cleansing their faces, the volunteers used the samples twice daily, once in the morning and once in the evening, for 4 weeks. Instrumental tests and investigator assessment methods were then used to assess the improvement, repair, and soothing effects of acne after the use of the blank essence and PM-NEs.

The TEWL was measured using the Delfin Vapometer transdermal water loss meter (Delfin company, Kuopio, Finland). The skin’s a* values were obtained using a Minolta chromatometer (CM 700 d, Minolta company, Osaka, Japan), whereas the skin’s red pigment value measurement was evaluated using a Mexameter MX18 (Courage-Khazaka Electronic, Cologen, Germany).

The investigator assessments, including an assessment of acne efficacy and a safety assessment, were conducted by two professional dermatologists. The acne efficacy assessment included the degree of inflammation of the facial lesions (a score of 0 means no redness, 9 means very red), the amount of non-inflammatory acne on the face (number of whiteheads and blackheads), and the amount of inflammatory acne on the face (number of red papules and pustules). The efficacy of the PM-NEs was evaluated by the change degree of each index in the test and control areas before and after use. All adverse skin reactions, such as skin erythema, tingling, itching, burning, etc., that occurred during the study were recorded and serious adverse reactions were reported promptly and were included as a safety assessment.

### 3.13. Statistical Analysis

All the in vitro test results are shown as mean ± SD from at least three independent experiments. A statistical analysis was performed with one-way ANOVA. The clinical trials data were analyzed by analysis of covariance (ANCOVA) with change (day 28 minus day 1) as the outcome, treatment as a factor, subjects as a random effect, and day 1 as a covariate. Comparisons of the key groups were calculated from the ANCOVA model. *p* < 0.05 was considered statistically significant.

## 4. Conclusions

In this study, novel NEs (PM-NEs) for transdermal co-delivery of skin barrier repair and anti-inflammatory active ingredients, such as paeonol and madecassoside, were successfully constructed. The prepared PM-NEs exhibited small and uniform nanoparticle size, high EE and LE, and good stability, as well as improved transdermal permeability and enhanced cellular uptake. In addition, the systematic study by cellular 3D skin models and human skin experiments not only illustrate the skin barrier repair and anti-inflammatory efficacy of the PM-Nes by enhancing the skin permeation of active ingredients, but also promoted the uptake of active ingredients by target cells with stronger effects than free actives ingredients. In conclusion, these results show that transdermal co-delivery nanocarrier technology can achieve synergistic effects of active ingredients with different mechanisms of action, which also provides a new strategy for the development of skin barrier repair and anti-inflammatory skincare products for sensitive skin.

## Data Availability

No new data were created or analyzed in this study. Data sharing is not applicable to this article.
